# Classifying the tumor immune microenvironment in cervical cancer based on nuclear cytoplasmic consistent genes

**DOI:** 10.1038/s41598-025-26740-4

**Published:** 2025-11-28

**Authors:** Jiawei Wang, Anji Chen, Qiuling Chen, Haoting Niu, Ying Mao, Xiang Hu, Shuanglin Xiang, Jun He

**Affiliations:** 1 Hunan Provincial Key Laboratory of Regional Hereditary Birth Defects Prevention and Control, Hunan Normal University Affiliated Changsha Hospital for Maternal and Child Health Care, Changsha, China; 2 State Key Laboratory of Developmental Biology of Freshwater Fish, College of Life Sciences, Hunan Normal University, Changsha, China; 3 Engineering Research Center for Antibodies From Experimental Animals of Hunan Province, College of Life Sciences, Hunan Normal University, Changsha, China

**Keywords:** Nuclear-cytoplasmic consistent genes, Cervical cancer, Tumor microenvironment, Single-cell and single-nucleus RNA sequencing, Prognostic biomarkers and risk stratification, Cancer, Computational biology and bioinformatics, Genetics, Immunology, Molecular biology, Biomarkers, Diseases

## Abstract

Treatment options for advanced or recurrent cervical cancer (CC) remain limited, highlighting the urgent need for effective molecular biomarkers and therapeutic strategies. This study investigates nuclear-cytoplasmic consistent genes (NCCGs) in CC and other epithelial-derived malignancies, exploring their potential in molecular subtyping, prognosis evaluation, and therapeutic response prediction. NCCGs were identified using single-cell sequencing and single-nucleus RNA sequencing data. Through Cox regression and single-sample gene set enrichment analysis, TCGA-CESC cohort were stratified into high-risk (HRG) and low-risk (LRG) groups. Pan-cancer analysis of 14 TCGA epithelial-derived malignancies, including BLCA and BRCA, validated the classification capability and prognostic relevance of NCCGs. Clinical utility in predicting chemotherapy and immunotherapy responses was assessed using GSE168009 and IMvigor210CoreBiologies cohorts. NCCGs effectively stratified CC cohort into HRG and LRG. LRG demonstrated significantly better survival (HR = 3.24, 95% CI 1.57–6.7) and higher immune scores, including elevated CD8^+^ T and memory CD4^+^ T cell levels. Pan-cancer analysis confirmed NCCGs’ ability to differentiate HRG and LRG and associate with overall survival. LRG also showed greater sensitivity to PD-1/CTLA4 inhibitors and chemotherapeutic agents (e.g., Panobinostat and Doxorubicin). The NCCG-based risk score showed robust accuracy in predicting chemotherapy and immunotherapy efficacy. This study reveals the molecular mechanisms and clinical significance of NCCGs in CC and epithelial-origin cancers. NCCGs hold value in molecular classification, prognostic assessment, and predicting therapeutic responses, offering new markers and targets for precision medicine.

**Supplementary Information:**

The online version contains supplementary material available at 10.1038/s41598-025-26740-4.

## Background

Cervical cancer (CC) is the fourth most common malignancy among women worldwide and poses a significant threat to women’s health^1^. In 2020, an estimated 604,127 new cases and 341,831 deaths from CC were reported globally^2^. The incidence of CC has declined significantly with improvements in the Human Development Index (HDI); however, the incidence in low-HDI countries remains three times higher than that in high-HDI countries, highlighting a pronounced socioeconomic disparity^2^. Cervical squamous cell carcinoma (SCC), accounting for approximately 75% of all cases, is the most common subtype and is strongly associated with high-risk human papillomavirus (HPV) infection^3^. Although the widespread implementation of HPV vaccination and screening technologies has markedly improved the prognosis of early-stage CC patients^4,5^, treatment options for advanced or recurrent CC remain limited. Moreover, the response rates to immunotherapy and targeted therapies are suboptimal^6–8^, underscoring the urgent need for the development of more effective molecular biomarkers and therapeutic strategies.

The exchange of materials and signaling between the nucleus and cytoplasm is a critical process for maintaining normal cellular functions. Previous studies have shown that nuclear-cytoplasmic transport is frequently disrupted in various cancers, affecting biological processes such as tumor growth, inflammatory responses, cell cycle regulation, and apoptosis^9,10^. However, research on nuclear-cytoplasmic dysfunction in CC remains largely unexplored, and most existing studies focus on whole-cell analyses, specifically lacking detailed investigation of specific cellular subpopulations.

Nuclear-cytoplasmic consistent genes (NCCGs) refer to those genes whose expression and function are simultaneously influenced by multiple regulatory mechanisms within both the nucleus and cytoplasm during tumor initiation and progression. This study is the first to investigate NCCGs in CC. These genes exhibit stable expression and are less likely to be influenced by external factors, suggesting their potential critical roles in cellular functions. Using single-cell sequencing (scRNA-seq) and single-nucleus RNA sequencing (snRNA-seq) techniques combined with TCGA cohort, we identified NCCGs from CC cohort and conducted molecular subtyping. The results showed that NCCGs effectively stratified CC cohort into HRG and LRG, which exhibited significant differences in immune infiltration characteristics, tumor mutation burden (TMB), and sensitivity to both immunotherapy and chemotherapy. Moreover, these genes demonstrated potential discriminatory power in other epithelial-origin cancers.

## Methods

### Data download and preparation

RNA sequencing (RNA-seq) data and somatic mutation data for TCGA-CESC, along with RNA-seq data for 14 epithelial-derived malignancies (including TCGA-HNSC, TCGA-BLCA, and TCGA-BRCA), were obtained using the TCGAbiolinks R package (version 2.28)^11^. The HPV^+^ TCGA-CESC cohort data were retrieved from previously published literature^6^. CC scRNA-seq data and chemoradiotherapy-related cohort were retrieved from the GEO database (https://www.ncbi.nlm.nih.gov/geo/), including GSE208653, GSE197461, GSE168652, and GSE168009. CC snRNA-seq data (SCP1950) were downloaded from the Single Cell Portal (https://singlecell.broadinstitute.org/single_cell). Additionally, an immunetherapy-related cohort for bladder cancer was accessed from the IMvigor210CoreBiologies R package (http://research-pub.gene.com/IMvigor210CoreBiologies).

### scRNA-seq and snRNA-seq data processing

The scRNA-seq and snRNA-seq data, which had undergone upstream processing (including quality control and genome alignment), were analyzed using the Seurat R package (version 4.3)^12^. Cells were filtered according to the following criteria: UMI counts > 1,000, UMI counts below the 97th percentile of total UMI counts, gene counts > 200, and mitochondrial UMI percentage < 10% (snRNA-seq: < 1%). Normalization of gene expression for each cell was performed using the “LogNormalize” method implemented in the NormalizeData function, where expression values were scaled by the total expression, multiplied by a scaling factor of 10,000, and log-transformed. Highly variable genes were identified using the FindVariableFeatures function, with the top 2000 genes retained for downstream analysis. The selected genes were then centered and scaled using the ScaleData function, and dimensionality reduction was performed using principal component analysis (PCA).

Based on the PCA results, a shared-nearest neighbour graph was constructed using the FindNeighbors function, and cells were clustered using the FindClusters function. To enhance visualization, the RunTSNE function was applied. Differentially expressed gene markers for each cluster were identified using the FindAllMarkers function, which compares gene expression in a given cluster with that of all other clusters. To mitigate technical artifacts, DoubletFinder R package (version 2.0)^13^ was used to identify and remove putative doublets, improving data quality. Batch effects across samples were corrected using the Harmony R package (version 1.2)^14^, ensuring effective integration and consistency across data.

### Cell type and gene annotation

The SingleR R package (version 2.2)^15^ was used to assign cell types to each cluster based on reference datasets. These initial annotations were subsequently validated and refined by comparing them with manually curated gene markers from published literature^16–20^, ensuring the accuracy and biological relevance of the identified cell clusters.

### Copy number alteration inference

The inferCNV R package (version 1.16, https://github.com/ broadinstitute/infercnv) was used to infer copy number variation (CNV) in the cell clusters. Genes were organized according to their chromosomal positions, and initial CNV were assessed based on gene expression levels, providing insights into genomic alterations within these cell populations.

### Cell stemness analysis

The runStemness function from the scCancer R package (version 2.2)^21^ was used to evaluate stemness scores for individual cells within cell clusters, providing a quantitative measure of their stem cell-like properties. This analysis facilitated the identification and characterization of cellular subpopulations with potential stem-like features.

### Differentially expressed gene

Differentially expressed genes (DEGs) between cell clusters in scRNA-seq and snRNA-seq data were identified using the FindMarkers function in the Seurat software. DEGs were selected based on the following criteria: expression in at least 25% of cells in either sample group, |log2FoldChange|> 0.25, and an adjusted *p* value < 0.05. For bulk RNA-seq data, DEGs were identified using the DESeq2 R package (version 1.40)^22^. The selection criteria were set to |log2FoldChange|> 1 and an adjusted *p* value < 0.05.

### Functional enrichment analyses

Functional enrichment analysis was performed using the clusterProfiler R package (version 4.8)^23^ to identify enriched Gene Ontology (GO) biological processes and Kyoto Encyclopedia of Genes and Genomes (KEGG) pathways^24,25^. Enrichment results with an adjusted *p* value < 0.05 were considered statistically significant.

Gene Set Enrichment Analysis (GSEA) was conducted using the gsea function, with gene sets achieving an adjusted *p* value < 0.05 considered as significantly enriched.

### Single-sample gene set enrichment analysis

Single-sample Gene Set Enrichment Analysis (ssGSEA) was performed using the GSVA R package (version 1.53)^26^. Risk-associated genes from NCCGs were used to distinguish between HRG and LRG.

### Iterative ranking-based gene set enrichment analysis

Iterative ranking-based gene set enrichment analysis (irGSEA) was conducted using the irGSEA R package (version 3.3)^27^ to compute differential pathways across cell clusters. The robust rank aggregation (RRA) algorithm implemented in the irGSEA package was used to compute differential pathways, providing a statistically robust method for identifying significant gene sets. Gene sets were obtained from the MSigDB database (https://www.gsea-msigdb.org/gsea/msigdb/) to ensure a comprehensive and high-quality resource for pathway enrichment analysis.

### Survival analysis

Kaplan–Meier survival curves were constructed using the survival R package (version 3.6) and the survminer R package (version 0.4)^28^ to visualize survival differences across groups within the cohort. Cox regression analysis was subsequently performed using the coxph function to identify risk-associated genes. Hazard ratios were calculated to quantify the relative risk, with genes showing a *p* value < 0.05 considered as significant risk factors.

### Somatic mutation analysis

Somatic mutation data for were analyzed using the maftools R package (version 2.16)^29^. Differentially mutated genes between HRG and LRG were identified, alongside the calculation of TMB for each group. Furthermore, differences in pathway-specific gene mutations were assessed to explore functional alterations associated with risk stratification.

### Tumor Microenvironment landscape

The stromal and immune (ESTIMATE) score were calculated using the ESTIMATE algorithm^30^ to characterize the tumor microenvironment (TME). To gain deeper insights into the immune infiltrating landscape, the abundance of tumor-infiltrating immune cells (TIICs) within the TME was assessed using the xCell R package (version 1.1)^31^ and the second module (Impute Cell Fractions) of the CIBERSORTx database (https://cibersortx.stanford.edu/). Additionally, the Tracking Tumor Immunophenotype (TIP) database (http://biocc.hrbmu.edu.cn/TIP/) was utilized to analyze the immune dynamics of cancer cohorts, providing a comprehensive understanding of the immune landscape within the TME.

### Risk model construction and validation

The risk model was constructed using the caret R package (version 6.0)^32^, with cohort-specific risk-associated genes as input parameters. In the GSE168009 cohort, patients with durable clinical benefit (DCB; progression-free period > 5 years) were classified as the LRG, while those with no durable benefit (NDB; progression-free period < 3 years) were classified as the HRG.

### Immunotherapy prediction

Immunophenoscore (IPS) data were obtained from The Cancer Immunome Atlas (TCIA, https://tcia.at/home), and tumor immune dysfunction and exclusion (TIDE) scores were retrieved from the TIDE (http://tide.dfci.harvard.edu) database. These datasets were integrated to comprehensively evaluate the potential therapeutic response of cancer samples to immune checkpoint inhibitors targeting PD-1 and CTLA-4.

### Chemotherapy prediction

The Genomics of Drug Sensitivity in Cancer (GDSC) database (https://www.cancerrxgene.org/), a widely utilized resource for identifying molecular markers of drug sensitivity in cancer, was employed to assess the drug response in CC samples. The half-maximal inhibitory concentration (IC50) values, a key indicator of drug efficacy, were calculated using the R package oncoPredict^33^, which enables systematic prediction of drug sensitivity based on transcriptomic data.

### Cell culture, RNA isolation, and quantitative real-time polymerase chain reaction

Two CC cell lines, HeLa and SiHa, were used in this study, both obtained from Zhongqiao Xinzhou Company. HeLa cells were cultured in Dulbecco’s Modified Eagle Medium (DMEM, Gibco, USA) supplemented with 10% fetal bovine serum (FBS) and 1% penicillin–streptomycin at 37 °C in a humidified incubator with 5% CO_2_. SiHa cells were cultured under identical conditions but in Minimum Essential Medium (MEM, Gibco, USA) supplemented with 10% FBS and 1% penicillin–streptomycin. When the cells reached approximately 90% confluence, both cell lines were seeded into 6-well plates and further cultured until the cell density exceeded 90%. Nuclear fractions of HeLa and SiHa cells were extracted using a nuclear extraction kit (Solarbio, China) following the manufacturer’s instructions, and the isolated nuclei were stored at − 80 °C for subsequent analysis. Total RNA was extracted from HeLa cells, SiHa cells, and their nuclear fractions using Trizol reagent (Takara, Japan). Complementary DNA (cDNA) was synthesized according to the protocol provided with the cDNA reverse transcription kit (Novoprotein, China). The specific primer sequences for target gene amplification are provided in the supplementary materials (Supplementary file 1, Table S1). Quantitative real-time polymerase chain reaction (qRT-PCR) was performed using a real-time fluorescence PCR system (ABI, USA), and data were analyzed accordingly.

### Statistical analysis

Statistical analyses and data visualization in this study were conducted using R software (version 4.3). Unless otherwise specified, continuous data were analyzed using the Wilcoxon test or Student’s *t* test. Survival differences between groups were assessed using the log-rank test. All statistical tests were two-tailed, and a *p* value < 0.05 was considered statistically significant across all analyses. Ns, *, **, *** and **** indicate not Significant, *p* < 0.05, *p* < 0.01, *p* < 0.001 and *p* < 0.0001, respectively.

## Results

### Construction of the scRNA-seq and snRNA-seq Atlas for CC

**Fig. 1:**
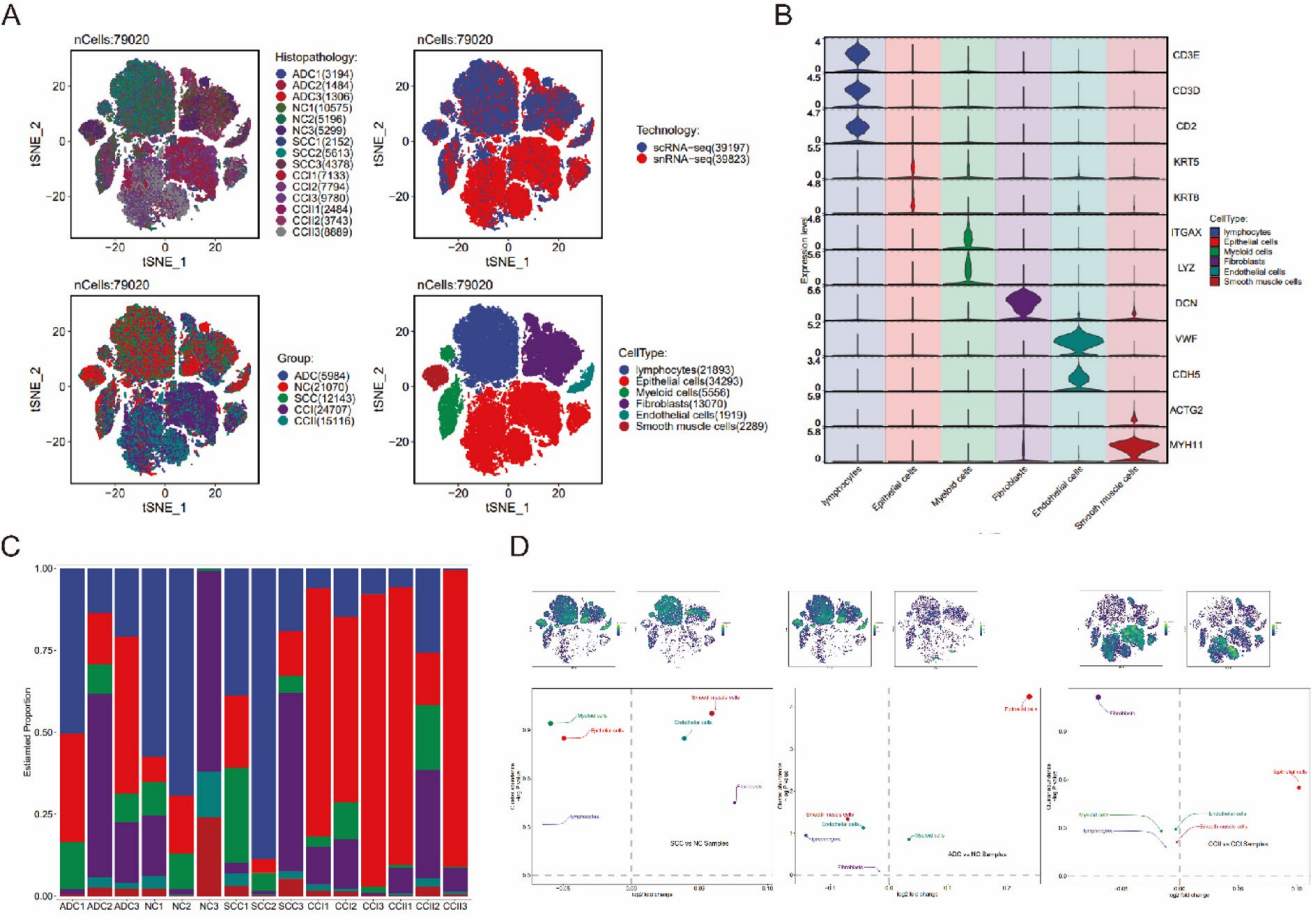
Tumor heterogeneity of CC at single-cell and single-nucleus resolution. (**A**) t-Distributed Stochastic Neighbor Embedding (t-SNE) plot showing 79,020 individual cells, colored by sample, sample group, technology type, and cell type distribution. Each point represents a single cell. (**B**) Violin plots depicting the marker genes for different cell types. (**C**) Proportional distribution of each cell type across different samples. (**D**) Volcano plots illustrating differential cell type distributions between SCC and NC, ADC and NC, and CCII and CCI. The density plots (top) display the t-SNE distribution of the corresponding sample groups.

We collected a total of 15 samples from public databases (Fig. 1A), including 3 SCC samples, 3 cervical adenocarcinomas (ADC) samples, and 3 HPV^+^ normal control (NC) samples analyzed through scRNA-seq, as well as 6 SCC samples (from CCI and CCII stages) analyzed through snRNA-seq. After stringent quality control, a total of 39,823 cells and 29,827 nuclei were identified. Based on the differential expression of characteristic marker genes, six cell clusters were manually annotated (Fig. 1B): lymphocytes (Lym) expressing CD3E, CD3D*,* and CD2; epithelial cells (Epi) expressing KRT5 and KRT8; myeloid cells (Mye) expressing *ITGAX* and LYZ; fibroblasts (Fb) expressing DCN; endothelial cells (Ec) expressing VWF and CDH5; and smooth muscle cells (Smc) expressing ACTG2 and MYH11.

Analysis of the proportional distribution of these cell clusters across the sample groups (Fig. 1C) revealed the presence of all six cell clusters in SCC, ADC, NC, CCI, and CCII samples, but with significant differences in their proportions. Pairwise comparisons of cell cluster proportions among the three groups (Fig. 1D) indicated a reduction in the proportion of lymphocytes in both SCC and ADC group compared to NC group. However, the remaining five cell clusters (Epi, Mye, Fb, Ec, Smc) showed opposing trends between SCC and ADC group, underscoring significant differences in cellular composition between these two cancer types.

Further analysis of CCI and CCII group revealed (Fig. 1D) a notable increase in the proportion of Epi in CCII group, consistent with the characteristic expansion of Epi during cancer progression. Conversely, Lym and Mye showed a significant decrease in CCII group, potentially reflecting immune suppression or immune evasion mechanisms. Additionally, the reduced proportions of Fb and Ec in CCII group may indicate impaired tissue repair capacity or abnormalities in vascular remodeling. The decrease in Smc could be associated with the degradation of vascular or smooth muscle structures. These findings highlight significant differences in cellular composition between different cancer types (SCC and ADC) and cancer progression stages (CCI and CCII), which may reflect the heterogeneity of the TME and its potential role in disease progression.

### Identification and functions of NCCGs in CC

**Fig. 2:**
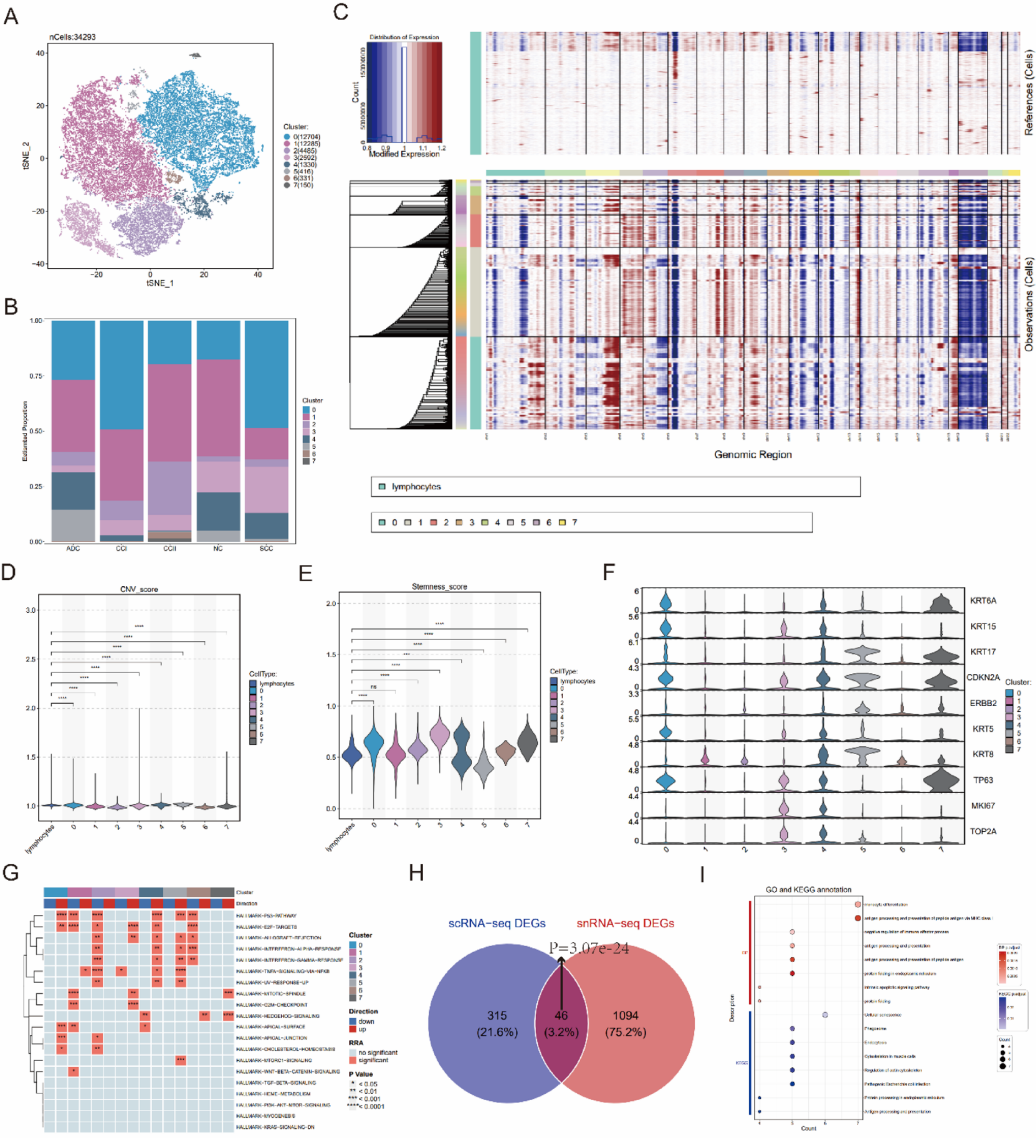
Identification and functional characterization of Epi and NCCGs. (**A**) t-SNE plot depicting 34,293 Epi, colored according to eight distinct Epi clusters. (**B**) Proportional distribution of each Epi cluster across different sample groups. (**C**) Heatmap showing large-scale CNVs in single cells and single nuclei, inferred from scRNA-seq and snRNA-seq data, with Lym treated as the reference (top). Large-scale CNVs are observed in the Epi clusters (bottom). (**D**) Violin plot comparing the CNV values between Lym cells and Epi clusters. (**E**) Violin plot comparing the stem cell scores between Lym cells and Epi clusters. (**F**) Violin plots depicting the marker genes for different Epi clusters. (**G**) Heatmap showing the top 20 HALLMARK pathways across Epi clusters. (**H**) Venn diagram illustrating the overlap of differential genes identified in cancerous Epi versus normal cells from scRNA-seq and snRNA-seq, revealing the NCCGs. (I) Bubble diagram depicting the top 8 KEGG pathways and GO biological processes (bp) terms identified in NCCGs.

Clustering analysis was performed on 34,293 Epi (Fig. 2A), resulting in the identification of eight distinct Epi subclusters. Most cells were distributed in clusters 0 and 1, while clusters 6 and 7 were specifically enriched in CCII group (Fig. 2B).

To confirm malignant Epi, CNV scores of Epi clusters relative to Lym were calculated using inferCNV analysis (Figs. 2C, D). Clusters 0, 3, 4, 5, and 7 exhibited higher CNV scores, with significant CNV alterations predominantly located in 1q, 3q, and 18q chromosomal regions. Stemness analysis (Fig. 2E) revealing that clusters 0, 3, 4, and 7 exhibited strong stemness characteristics, which are closely associated with tumor cell proliferation and survival advantages. Additionally, genes associated with malignant Epi, including KRT6A*,* KRT15*,* KRT17*,* CDKN2A*,* and ERBB2, were highly expressed in clusters 0, 3, 4, 5, and 7 (Fig. 2F). irGSEA analysis of the HALLMARK pathways (Fig. 2G) showed that the p53 signaling pathway, closely linked to cancer progression, was significantly upregulated in clusters 0, 4, and 5. The mTORC1 signaling pathway, which regulates metabolism and cell growth, was highly expressed in cluster 5. Additionally, pathways associated with tumor proliferation and cell cycle regulation exhibited distinct patterns among clusters. Specifically, E2F targets were highly expressed in clusters 0, 3, and 4; G2M checkpoint genes were primarily upregulated in cluster 3; and the mitotic spindle pathway was prominently expressed in clusters 3 and 7. These findings indicate that cluster 3 exhibited comprehensive activation of cell cycle pathways, suggesting enhanced proliferative and division capacity, while clusters 0, 4, 5, and 7 displayed unique pathway characteristics. The high expression of proliferation-related genes (MKI67 and TOP2A) in clusters 3, 4, and 5 further supports these observations. Integrating results from multiple analyses, clusters 0, 3, 4, 5, and 7 were ultimately identified as malignant Epi clusters.

Differential gene expression analysis was performed by comparing single-cell and single-nucleus malignant Epi with their normal epithelial counterparts. This analysis identified 361 and 1,140 DEGs (Supplementary file 2, Table S2, S3), respectively, with 46 overlapping genes defined as NCCGs (Fig. 2H, Chi-Square Test < 0.05, Supplementary file 2, Table S4). GO functional enrichment analysis (Fig. 2I) revealed that these genes were significantly involved in three major biological processes: protein processing and homeostasis regulation (e.g., “protein folding” and "protein folding in the endoplasmic reticulum"), immune-related functions (e.g., "antigen processing and presentation" and “negative regulation of immune effector process”), and cellular differentiation and apoptosis (e.g., “monocyte differentiation” and "intrinsic apoptotic signaling pathway").

KEGG pathway analysis (Fig. 2I) further demonstrated that these genes participated in several key pathways, including protein processing and folding, cytoskeletal dynamics (e.g., " Cytoskeleton in muscle cells " and "Regulation of actin cytoskeleton "), immune regulation, and cellular senescence (e.g., "antigen processing and presentation" and “cellular senescence”). Interestingly, certain pathways (e.g., "pathogenic Escherichia coli infection") may influence CC development and progression by modulating the TME and inflammatory signaling.

Collectively, these findings highlight the critical roles of NCCGs in immune responses, protein homeostasis, cytoskeletal dynamics, and cellular senescence, shedding light on their potential contributions to CC pathogenesis.

## Expression and prognostic significance of NCCGs in CC

**Fig. 3:**
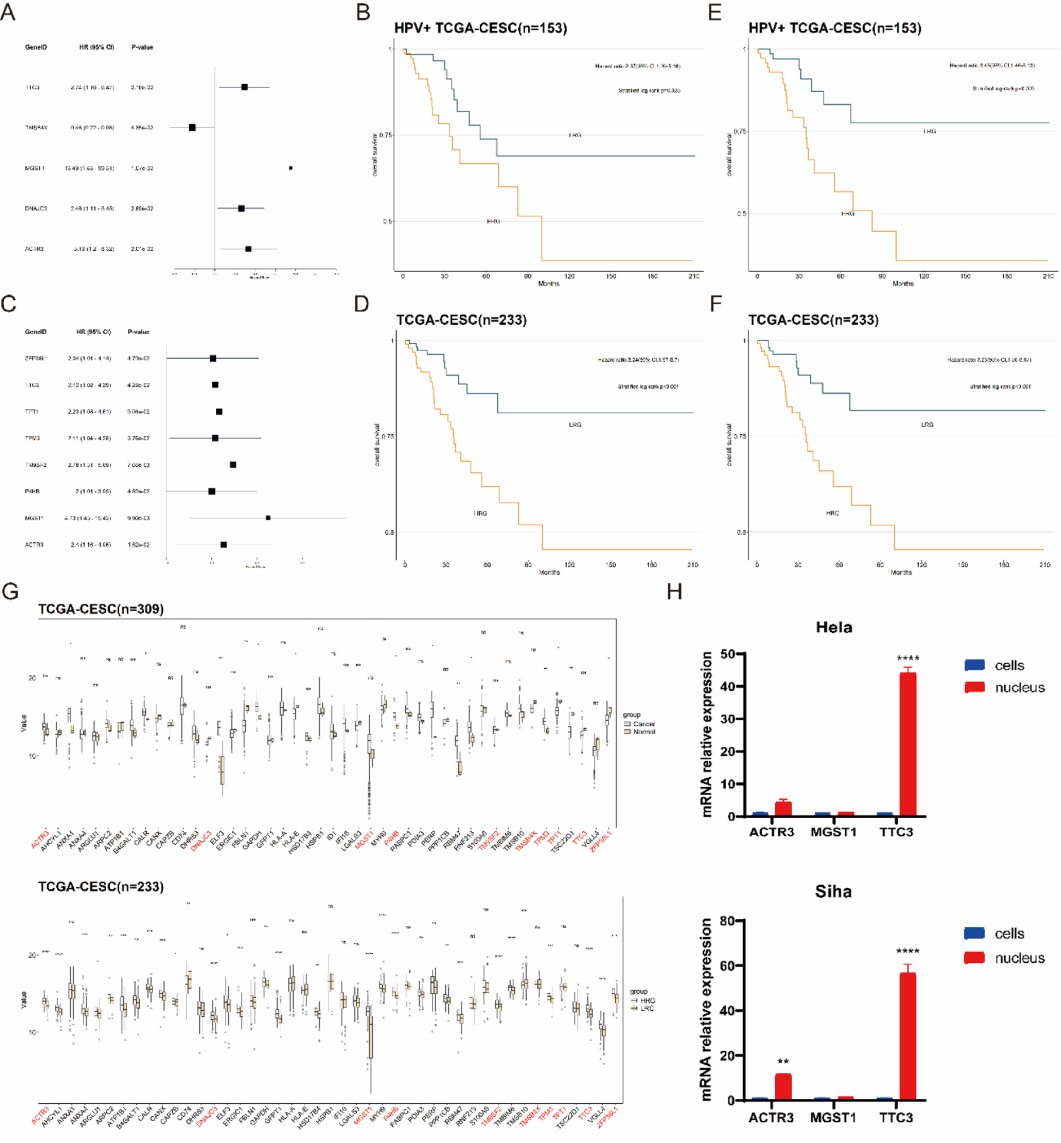
Identification of NCCGs expression patterns in CC. (**A**, **C**) Forest plots showing NCCGs associated with OS in the HPV^+^ TCGA-CESC cohort (**A**) and the TCGA-CESC cohort (**C**). (**B**, **D**–**F**) Kaplan–Meier survival curves comparing OS between HRG and LRG in the HPV^+^ TCGA-CESC cohort (**B**, **E**) and the TCGA-CESC cohort (**D**, **F**). (**G**) Box plots showing the differential expression of NCCGs between CC and NC group in the TCGA-CESC cohort (top), and between HRG and LRG (bottom). (**H**) Bar chart depicting the relative expression levels of TTC3, MGST1, and ACTR3 in the nucleus and whole cells of Hela and Siha cell lines.

To explore the biological significance of NCCGs in CC, we first performed Cox regression analysis in the HPV^+^ TCGA-CESC cohort (n = 153). Genes significantly associated with overall survival (OS) were identified (Fig. 3A, Supplementary file 3, Figure S1), including TTC3 (HR = 2.74, 95% CI 1.16–6.47), TMSB4X (HR = 0.46, 95% CI 0.22–0.98), MGST1 (HR = 13.49, 95% CI 1.83–99.31), DNAJC3 (HR = 2.46, 95% CI 1.11–5.45), and ACTR3 (HR = 3.16, 95% CI 1.20–8.32). Based on ssGSEA scores derived from these five genes, patients were stratified into HRG and LRG. Survival analysis (Fig. 3B) revealed that HRG had significantly poorer survival compared to LRG (HR = 2.37, 95% CI 1.09–5.16).

To validate the universality of the biological significance of NCCGs, we repeated the analysis (Fig. 3C, D, Supplementary file 3, Figure S2) in the complete TCGA-CESC cohort (n = 233). Similarly, LRG exhibited significantly better survival outcomes than HRG (HR = 3.24, 95% CI 1.57–6.7). Further analysis identified eight genes significantly associated with OS, including ZFP36L1 (HR = 2.04, 95% CI 1.01–4.14), TTC3 (HR = 2.12, 95% CI 1.02–4.39), TPT1 (HR = 2.23, 95% CI 1.08–4.61), TPM3 (HR = 2.11, 95% CI 1.04–4.28), TM9SF2 (HR = 2.78, 95% CI 1.31–5.89), P4HB (HR = 2.00, 95% CI 1.01–3.99), MGST1 (HR = 4.73, 95% CI 1.45–15.42), and ACTR3 (HR = 2.40, 95% CI 1.16–4.96). A comparison of the risk genes between the two cohorts revealed that TTC3, MGST1, and ACTR3 were consistently associated with OS in both cohorts. ssGSEA analysis (Fig. 3E, F) based on these three genes yielded consistent results, further demonstrating their strong ability to distinguish HRG from LRG.

We also analyzed the expression of NCCGs in CC cohorts (Fig. 3G). Compared with NC group, only a small number of NCCGs, such as ANXA1, TPM3, and TPT1, exhibited significant differential expression in CC group. However, nearly 70% of these genes showed significant differences between HRG and LRG, indicating strong inter-gene associations, even though some may not be direct risk factors for CC.

To further validate the expression of NCCGs in both whole cells and nuclei, we used the Human Protein Atlas (HPA, https://www.proteinatlas.org/) database to analyze the similarity in gene expression between the TCGA-CESC cohort and CC cell lines. The analysis revealed that HeLa and SiHa cell lines exhibited 75% and 77% similarity, respectively, to the TCGA-CESC cohort. Thus, these two cell lines were selected for experimental validation. qRT-PCR analysis (Fig. 3H) showed that TTC3, MGST1, and ACTR3 were expressed in both the nuclei and whole cells. Notably, the nuclear expression of TTC3 in HeLa cells was significantly higher compared to its expression in whole cells. In SiHa cells, the nuclear expression levels of ACTR3 and TTC3 were also significantly higher than their corresponding levels in whole cells.

In summary, the expression patterns of TTC3, MGST1, and ACTR3 were highly consistent across the two TCGA-CESC cohorts and CC cell lines, highlighting their critical role in OS among CC patients. These findings lay a solid foundation for further investigation into their underlying mechanisms and potential clinical applications.

## Immunological characteristics of the LRG reveal antitumor activity and TME advantages

**Fig. 4:**
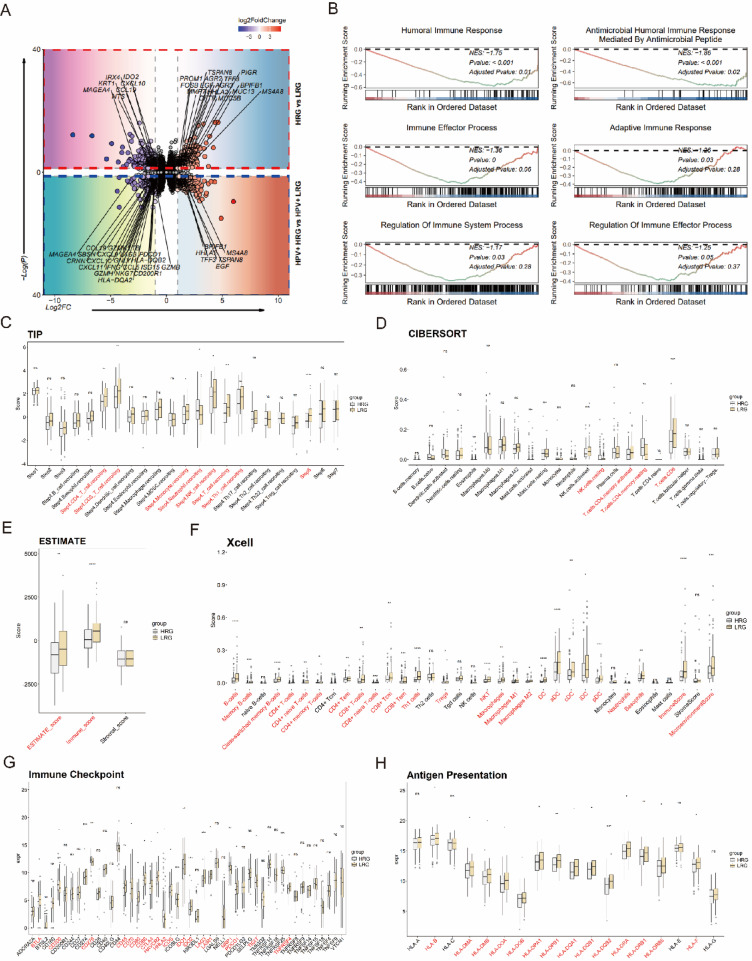
Relationship between NCCG clusters and the TME. (**A**) Volcano plot showing the distribution of DEGs between HRG and LRG in the HPV^+^ CESC cohort and the TCGA-CESC cohort. (**B**) GSEA plot illustrating the enrichment of immune-related GO terms in DEGs between HRG and LRG. (**C**) Box plot showing differences in cancer immune cycle step scores between HRG and LRG. (**D**, **F**) Box plots showing differences in the enrichment of TIICs as assessed by CIBERSORT (**E**) and Xcell (**F**) between HRG and LRG. (**E**) Box plot showing differences in stromal score, immune score, and ESTIMATE score between HRG and LRG. (**G**, **H**) Box plots showing differences in the expression of immune checkpoint genes (**G**) and antigen-presenting genes (**H**) between HRG and LRG.

To investigate the differences between the HRG and LRG, differential gene expression analysis (Fig. 4A) was performed on the HPV^+^ TCGA-CESC cohort and the TCGA-CESC cohort. A total of 591 and 609 DEGs were identified (Supplementary file 2, Table S5, S6), respectively. In the HRG, tumor suppressor genes such as BPIFB1 and MS4A8, as well as hypoxia response-related genes like EGF, were significantly upregulated. In contrast, the LRG demonstrated significant upregulation of pro-inflammatory genes (FOSB, LTB, CXCL10, CXCL11, CXCL9, CCL5), immune checkpoint genes (except HHLA2, such as IDO2, PDCD1, LAG3, and CD200R1), antigen presentation genes (HLA-DQB2, HLA-DQA2), T cell cytotoxicity-related genes (NKG7, GZMH, GZMK, GNLY, GZMB, IFNG), T cell exhaustion markers (LAG3), and immunosuppressive T cell-associated genes (CCL19). These findings suggest that the LRG exhibits stronger pro-inflammatory and immune activity features.

Subsequent analyses were all conducted based on the TCGA-CESC cohort. GSEA analysis further validated these findings (Fig. 4B), showing significant downregulation of immune-related pathways (e.g., “humoral immune response” and “adaptive immune response”) in the HRG, indicating reduced immune activity in this group.

We further evaluated the differences in tumor immunological characteristics and TME between the two groups. Cancer immunity cycle analysis (Fig. 4C) revealed that the LRG had significantly higher scores in immune cell trafficking to the tumor (Step 4), including migration of CD4^+^ T cells, CD8^+^ T cells, monocytes, NK cells, Th1 cells, and T cells, while neutrophil migration scores were significantly lower. Similarly, the LRG exhibited higher scores in immune cell infiltration into tumors (Step 5). Consistent with these findings, the LRG also showed elevated immune and ESTIMATE scores (Fig. 4E). XCell and CIBERSORT analyses (Fig. 4D, F) further revealed significantly increased levels of TIICs in the LRG, particularly CD8^+^ T cells and CD4^+^ memory T cells, with a corresponding increase in ImmuneScore further supporting these observations.

We also analyzed the expression of immune checkpoint and antigen presentation genes (Fig. 4G, H) in the two groups. In the LRG, immune checkpoint genes such as BTLA, CD48, CD80, CTLA4, and PDCD1 were significantly upregulated, while CD200 and HHLA2 were downregulated. Antigen presentation genes also showed an overall upregulation trend in the LRG, with significant increases in HLA-B, HLA-DPA1, HLA-DPB1, HLA-DQB1, and HLA-DRB1 expression.

In summary, these findings suggest that the LRG is characterized by stronger immune activity, higher levels of TIICs, and more robust antigen presentation and immune checkpoint gene expression profiles. These features collectively highlight the superior antitumor immune response and favorable TME observed in the LRG.

### Association of NCCGs with genomic mutation characteristics

**Fig. 5:**
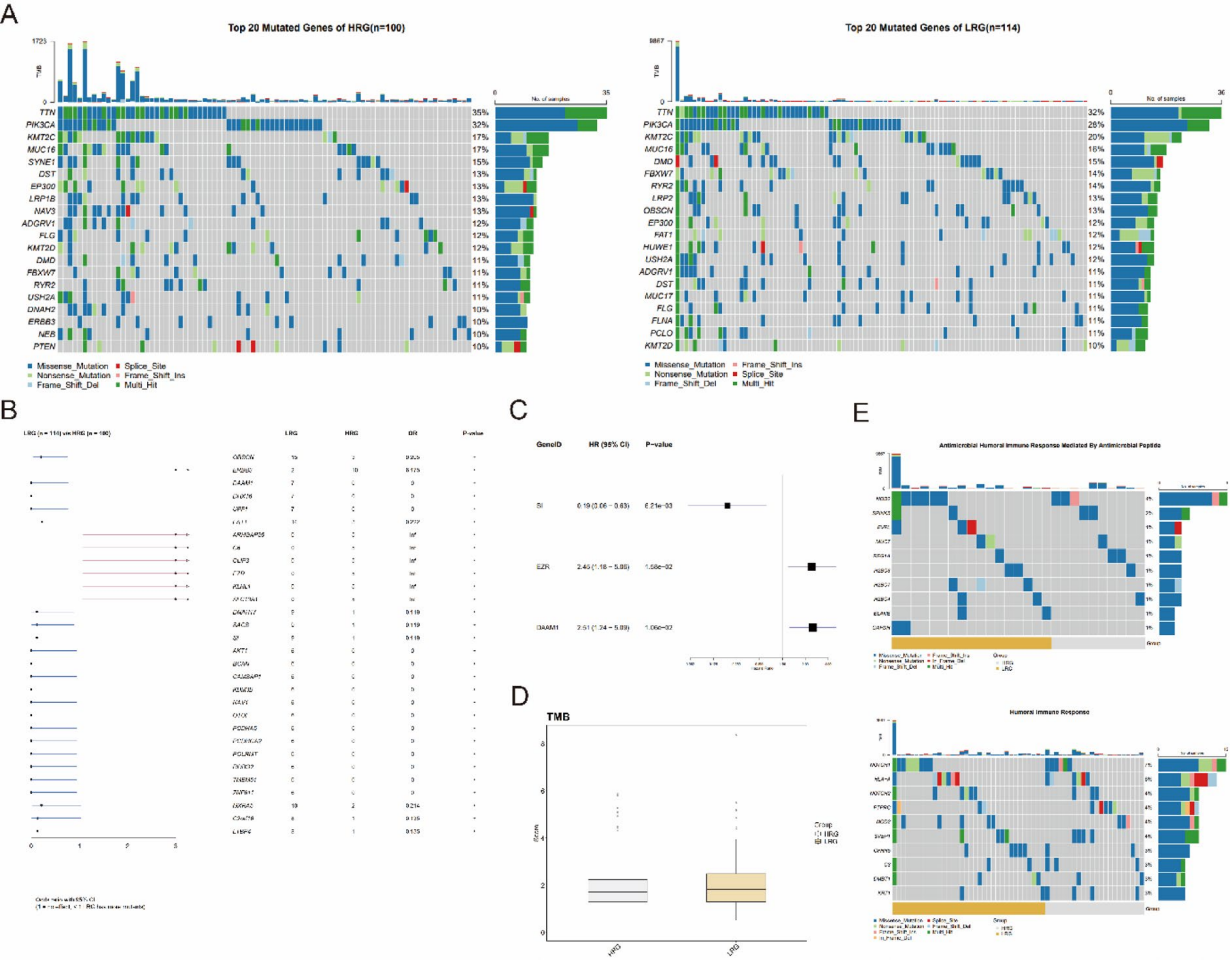
Genomic alterations in CC associated with NCCG clusters. (**A**) Waterfall plot showing the top 20 mutated genes between HRG and LRG. (**B**, **C**) Forest plots depicting differential mutated genes between HRG and LRG (**B**) and those associated with OS in the TCGA-CESC cohort (**C**). (**D**) Box plot showing differences in TMB between HRG and LRG. (**E**) Waterfall plot displaying the differences in immune-related GO term gene mutations between HRG and LRG.

To explore the genomic mutation differences between the HRG and LRG, we conducted a somatic mutation analysis and found 35% of the top 20 mutated genes differed between the two groups (Fig. 5A). Then, we compared the gene mutation frequencies between the two groups (Fig. 5B) and identified 30 genes with significantly different mutation frequencies. Among them, seven genes were specific to the HRG. Using Cox regression analysis (Fig. 5C), we evaluated the relationship between these mutated genes and OS. Three genes showed significant associations with OS: SI (HR = 0.19, 95% CI 0.06–0.63), EZR (HR = 2.45, 95% CI 1.18–5.06), and DAAM1 (HR = 2.51, 95% CI 1.24–5.09).

TMB is closely linked to immune cell infiltration and immune activity. Although the LRG exhibited a slightly higher overall TMB compared to the HRG (Fig. 5D), the difference was not statistically significant. Furthermore, no significant correlation was observed between TMB and key NCCGs (TTC3, MGST1, and ACTR3). However, an analysis of immune-related GO term mutations (Fig. 5E) revealed that the LRG harbored a higher number of immune-related gene mutations compared to the HRG. This included a greater frequency of mutations in genes associated with immune activity and OS.

In summary, despite the lack of significant differences in overall TMB between the HRG and LRG, the LRG exhibited a higher frequency of immune-related gene mutations and included more OS-associated mutated genes. These findings suggest that the LRG may exhibit stronger immune activity, potentially contributing to its improved prognosis compared to the HRG.

### Predictive role of NCCGs in tumor immunotherapy and chemotherapy response

**Fig. 6:**
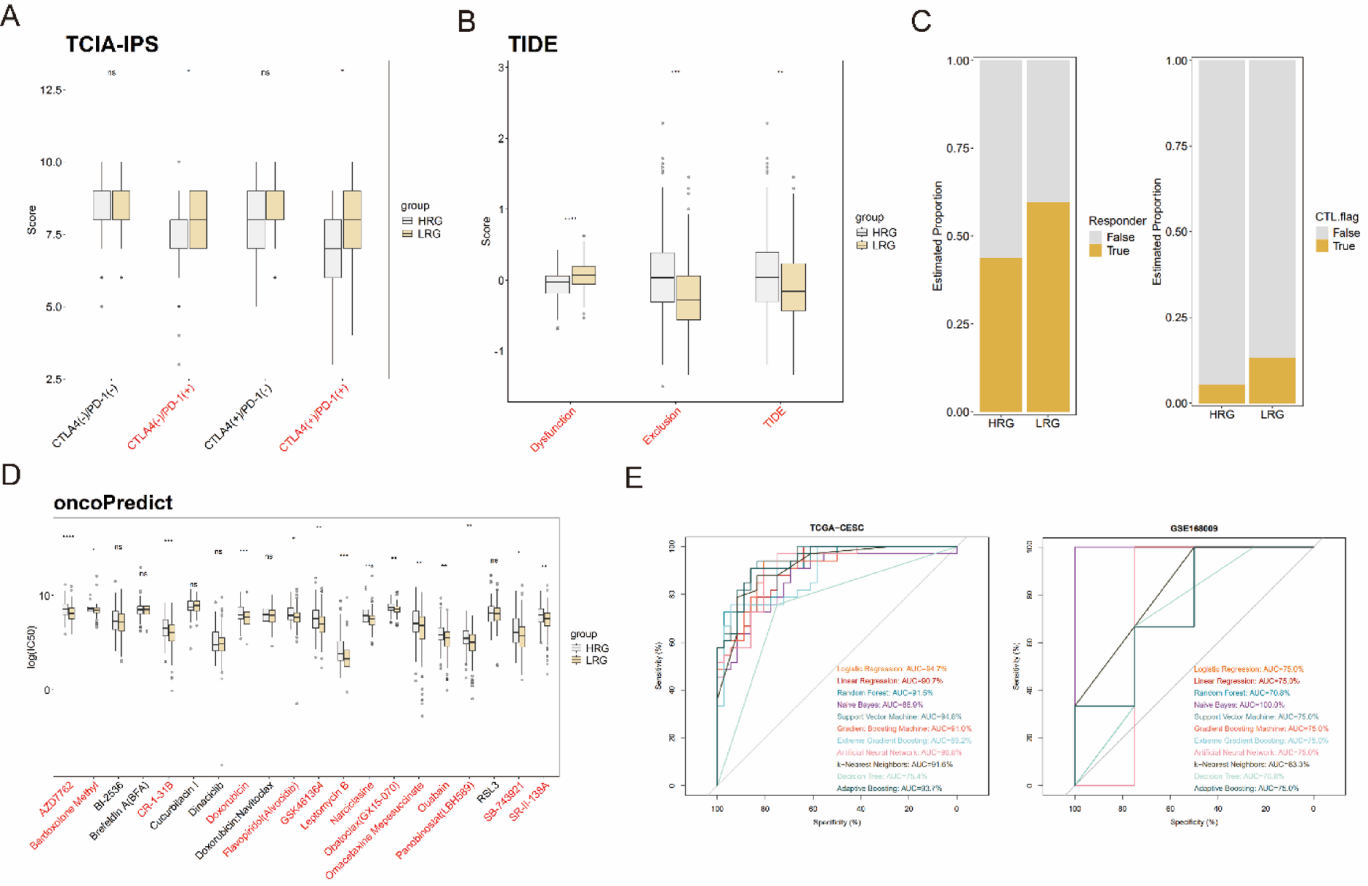
Immune and chemotherapy risk prediction and model construction based on NCCG clusters. (**A**, **B**) Box plots showing differences in IPS scores (A) and Dysfunction, Exclusion, and TDE scores (**B**) between HRG and LRG. (**C**) Stacked bar chart illustrating the predicted differences in ICI responders, and CTLs between HRG and LRG. (**D**) Box plot showing differences in the predicted IC50 values for the top 20 drugs between HRG and LRG. (**E**) ROC curves and AUC values for different algorithm models in the TCGA-CESC and GSE168009 cohorts.

We extracted the IPS of the HRG and LRG from the TCIA database. The results showed that LRG had significantly higher IPS for PD-1 inhibitors and combined PD-1 and CTLA4 inhibitors compared to the HRG (Fig. 6A). Additionally, predictions from the TIDE database supported this trend. The proportion of immune checkpoint inhibitor (ICI) responders was significantly higher in the LRG, and these patients exhibited superior cytotoxic T lymphocyte (CTL) infiltration levels compared to the HRG (Fig. 6C). However, the LRG demonstrated higher T-cell dysfunction scores, while the HRG had significantly elevated T-cell exclusion scores and overall TIDE scores (Fig. 6B). These findings suggest that LRG may have a higher potential response to ICI therapy.

We then utilized the oncoPredict R package to predict chemotherapy drug sensitivity in the two groups (Fig. 6D) and identified representative drugs with lower IC50 values. The results revealed that LRG were significantly more sensitive to several classical chemotherapeutic agents, including Panobinostat (LBH589), Doxorubicin, Flavopiridol (Alvocidib), Bardoxolone Methyl, AZD7762, and Obatoclax (GX15-070), compared to HRG. These drugs, supported by existing clinical or experimental studies, represent promising candidates for further investigation into precise treatment strategies.

Furthermore, we developed a chemotherapy risk prediction model using TTC3, MGST1, and ACTR3 as parameters (Fig. 6E). With 70% of the TCGA-CESC cohort used as a training set, we applied logistic regression, linear regression, random forest, naive Bayes, and other algorithms to construct the model. The model was validated on the remaining 30% of the TCGA-CESC cohort and an external cohort, GSE168009. The naive Bayes algorithm demonstrated the best predictive performance, achieving AUC values consistently above 86% across all datasets. This indicates high robustness and potential clinical utility of the model in predicting chemotherapy response.

In summary, our findings suggest that LRG exhibit superior responsiveness to immunotherapy and chemotherapy, while the proposed predictive model based on NCCGs shows promise for guiding precision treatment in CC.

### Predictive role of NCCGs in epithelial-derived malignancies

**Fig. 7:**
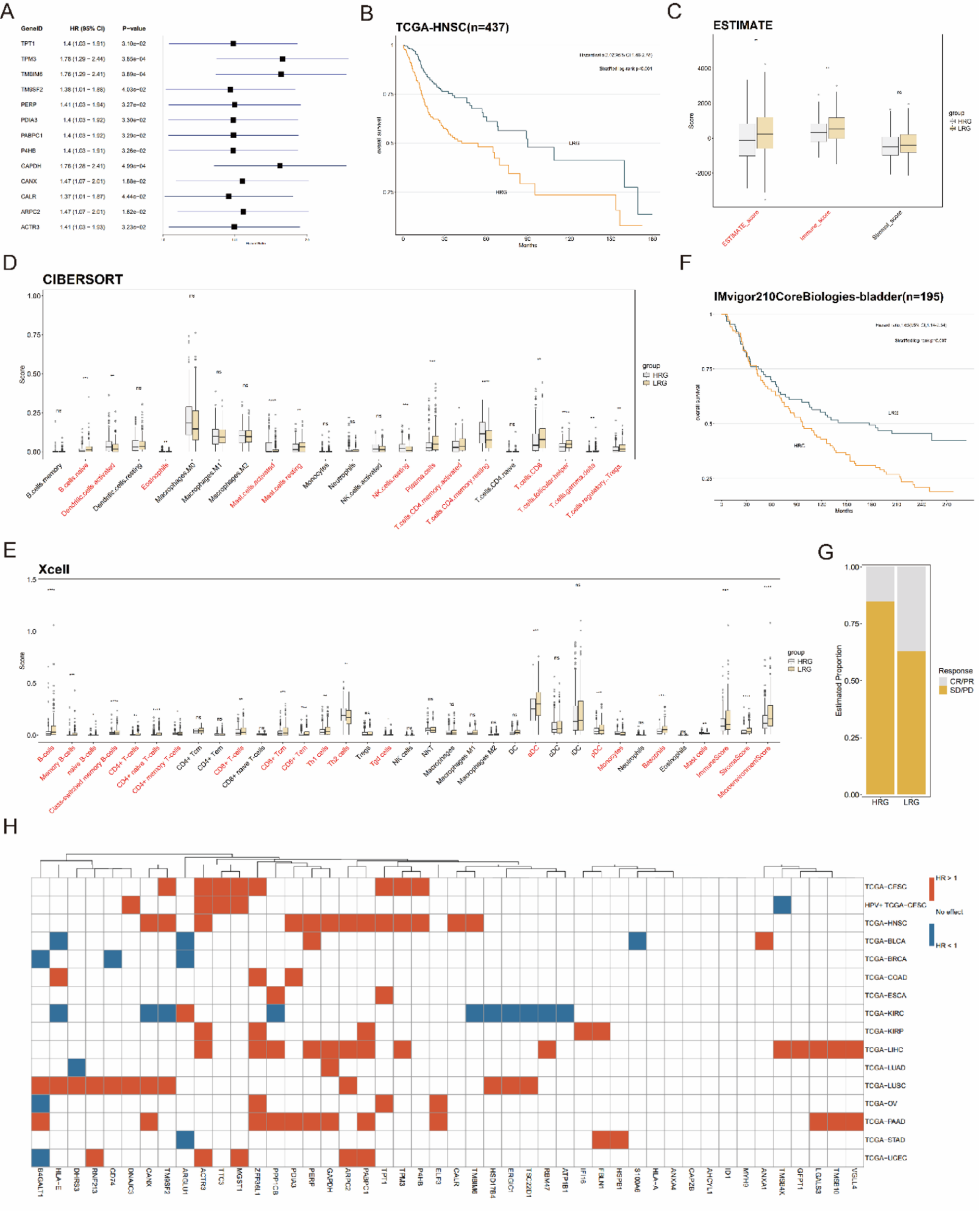
Identification of NCCG expression patterns in other epithelial-derived malignancies. (**A**) Forest plots showing NCCGs associated with OS in the TCGA-HNSC cohort. (**B**) Kaplan–Meier survival curves comparing OS between HRG and LRG in the TCGA-HNSC cohort. (**C**) Box plot showing differences in stromal score, immune score, and ESTIMATE score between HRG and LRG. (**D**, **E**) Box plots showing differences in the enrichment of TIICs as assessed by CIBERSORT (**D**) and xCELL (**E**) between HRG and LRG. (**F**) Kaplan–Meier survival curves comparing OS between HRG and LRG in the IMvigor210CoreBiologies-bladder cohort. (**G**) Stacked bar chart showing the proportion of CR/PR between HRG and LRG as predicted by the model. (**H**) Heatmap displaying NCCGs associated with OS in other epithelial-derived malignancies.

To evaluate the prognostic value of NCCGs in HPV-associated cancers, we performed Cox regression analysis in the TCGA-HNSC (n = 437) cohort (Fig. 7A). The results identified 13 genes significantly associated with OS. Among these, TM9SF2, ACTR3, TPT1, TPM3, and P4HB overlapped with the risk genes identified in the TCGA-CESC cohort, all showing HR values greater than 1, further supporting their critical role in HPV-associated cancers.

Based on ssGSEA scores calculated from these 13 genes, patients were stratified into HRG and LRG. Survival analysis (Fig. 7B) revealed that the LRG exhibited significantly better survival compared to the HRG (HR = 2.02, 95% CI 1.46–2.78). Further investigation of the tumor immunological characteristics and TME differences between the two groups showed that the LRG had significantly higher immune scores and ESTIMATE composite scores than the HRG (Fig. 7C). This finding was validated through CIBERSORT analysis. Additionally, XCell and CIBERSORT analyses (Fig. 7D, E) indicated that TIICs, including CD8^+^ T cells, CD4^+^ memory T cells, and B cells, were significantly upregulated in the LRG. These results suggest that the LRG is associated with stronger antitumor immune activity, further emphasizing the predictive value of NCCGs in HPV-associated cancers.

To assess the prognostic utility of NCCGs in other epithelial-derived malignancies, we extended the analysis to 13 TCGA cancer cohorts, including TCGA-BLCA, TCGA-BRCA, and TCGA-COAD, employing the same methodology (Fig. 7H). The results demonstrated that these genes could robustly stratify patients into HRG and LRG across various cancer types, with the LRG consistently showing higher levels of immune infiltration. Additionally, certain genes, such as B4GALT1, HLA-E, and PABPC1, were found to be significantly associated with OS in multiple cancers, suggesting their pivotal roles in immune regulation across different cancer types.

To further validate the predictive capability of NCCGs, we performed an independent validation using the IMvigor210CoreBiologies R package bladder cancer cohort (n = 195). The results confirmed the findings (Fig. 7F, G) from the TCGA-BLCA cohort: LRG had a significantly higher proportion of complete response/partial response (CR/PR) to immunotherapy and improved survival compared to HRG (HR = 1.63, 95% CI 1.14–2.34). These findings indicate that immune risk scores based on NCCGs not only effectively stratify prognostic risk across various cancer types but also distinguish treatment efficacy in immunotherapy cohorts, highlighting their potential for clinical application.

## Discussion

The boundary region between different Epi types (e.g., squamous epithelium and columnar epithelium), known as the transitional zone, is characterized by unique biological properties, making it particularly susceptible to infections and tumorigenesis. It is also considered a potential source of cancer-initiating cells^34,35^. In this study, we identified five malignant Epi clusters through preliminary analyses. Among them, clusters 4, 5, and 7 exhibited high expression of both the squamous epithelial marker KRT5 and the columnar epithelial marker KRT8, reflecting molecular characteristics consistent with the transitional zone. Additionally, clusters 0, 3, 4, and 7 showed high expression of KRT5 and TP63, suggesting that these cells may originate from the primitive squamous cell lineage of the ectocervix^16^. Notably, clusters 4 and 5 demonstrated significantly upregulated expression of proliferation-associated genes, indicating a high proliferative potential. Based on these findings, we hypothesize that clusters 4 and 5 represent the cancer-initiating cells of CC. Furthermore, we propose that the differentiation trajectory of malignant squamous cell clusters likely follows the path of 4-7-0/3. This study provides new insights into the mechanism underlying the origin of CC and highlights the critical role of the transitional zone in tumorigenesis.

In the HSIL (high-grade squamous intraepithelial lesion) stage of CC, a transient amplifying cell, which exists in an intermediate state between cancer stem cells and their derived tumor cells, has been identified^36–38^. In this study, through in-depth characterization of malignant Epi, we identified similar cell clusters in NC group. These clusters were closely associated with clusters 0, 3, 4, and 5, suggesting a potential connection with malignant Epi. This finding provides critical insights into the key molecular events underlying CC precancerous lesions and suggests that HPV infection may promote lesion progression by enhancing stemness or conferring a proliferative advantage to certain cells within the microenvironment, thereby driving disease progression.

Using Cox regression and ssGSEA analyses of the TCGA-CESC and HPV^+^ TCGA-CESC cohorts, this study identified 10 NCCG-based risk genes (TTC3, TMSB4X, MGST1, DNAJC3, ACTR3, ZFP36L1, TPT1, TPM3, TM9SF2, and P4HB). Among these, TTC3, a critical E3 ubiquitin ligase, promotes cell cycle progression and tumor cell proliferation in gastric cancer by mediating the ubiquitination and degradation of 14–3-3σ^39^. In cardiac tissue, circ-Ttc3 protects cardiomyocytes from apoptosis through miRNA regulatory mechanisms^40^. TMSB4X functions as a multifunctional regulatory factor, playing a crucial role in CC by modulating the cytoskeleton and autophagy. Additionally, in non-small cell lung cancer, TMSB4X promotes immune evasion by regulating dendritic cell activity and the tumor immune microenvironment^41,42^. MGST1, which possesses glutathione transferase and peroxidase activities, plays a multifaceted role in tumorigenesis, particularly by influencing cancer cell survival and proliferation through antioxidant, anti-apoptotic, and drug-resistance mechanisms^43^. DNAJC3, an important member of the heat shock protein family, exhibits bidirectional regulatory effects and plays a role in tumor cell proliferation, apoptosis, autophagy, and drug resistance. Its regulation by miRNAs and non-coding RNAs makes it a significant gene for studying treatments for CC and other cancers^44–47^. ACTR3 has been identified as a potential biomarker in oral squamous cell carcinoma^48^. ZFP36L1, an RNA-binding protein, suppresses the cell cycle by degrading mRNAs such as Cyclin D1, thereby exerting tumor-suppressive effects. It also regulates immune factors, including TNF-α and VEGF-A, influencing the TME. The loss of ZFP36L1 enhances drug resistance, highlighting its potential therapeutic value in cancer treatment^49–51^. TPT1, a pro-oncogenic protein, promotes proliferation, migration, and invasion in CC cells by regulating the PI3K/AKT signaling pathway and epithelial-mesenchymal transition. Both TPT1 and its antisense RNA TPT1-AS1 may play critical roles in CC progression, with significant diagnostic and therapeutic potential^52–54^. TPM3 is highly expressed in CC and plays a dual role in promoting tumor progression. It not only facilitates cell proliferation, migration, and invasion but also regulates the immune microenvironment through pathways such as PI3K/AKT. The downregulation of miR-377-5p, which targets TPM3, exacerbates tumor malignancy, making TPM3 a promising diagnostic and therapeutic target^55,56^. TM9SF2 is highly expressed in colorectal cancer and is associated with tumor invasion, metastasis, and poor prognosis^57^. P4HB is overexpressed in various cancers and is involved in collagen metabolism, hypoxic responses, and TME regulation. Its high expression is associated with advanced tumor stages and poor prognosis. P4HB promotes tumor growth and migration, making it a potential diagnostic and therapeutic target in CC^58–60^. In summary, these risk genes exhibit diverse oncogenic or tumor-suppressive functions across multiple cancers, suggesting their involvement in CC development and progression through mechanisms such as cell cycle regulation, signaling pathways, autophagy, and the immune microenvironment. Notably, genes such as TTC3, TMSB4X, MGST1, DNAJC3, and P4HB are closely linked to cancer cell proliferation and migration and may serve as key regulators of CC progression. Meanwhile, genes like TPM3 and TPT1 exhibit specific oncogenic roles in CC, particularly influencing invasion and metastasis via critical signaling pathways such as PI3K/AKT. The overexpression or specific regulatory functions of these genes provide new perspectives on the molecular mechanisms of CC and offer potential directions for the development of diagnostic biomarkers and targeted therapies.

CC exhibits significant heterogeneity in both clinical manifestations and molecular characteristics. The team led by Xiaojun^61^ performed clustering analyses on HPV^+^ CC cohort and identified two subtypes: HPV^+^G1 and HPV^+^G2. HPV^+^G1 was characterized by higher immune infiltration levels, greater stromal content, and better disease-free survival outcomes, while HPV^+^G2 exhibited lower tumor purity and higher stemness scores. In our differential analysis of HRG and LRG, we observed that highly expressed genes such as MUC13, TFF3, PIGR, MUC5B, AGR3, TSPAN8, and PROM1 overlapped significantly with marker genes of the HPV^+^G1 subtype. Conversely, highly expressed genes in the HRG, including MAGEA4, KRT1, NTS, IRX4, CRNN, and SBSN, were consistent with certain molecular features of the HPV^+^G2 subtype. Additionally, the teams led by Li Jun^62^ and Jin^63^ independently performed clustering analyses of CC cohort based on ferroptosis-related genes and double-stranded RNA-binding proteins, respectively, to identify molecular subtypes. These subtypes exhibited differences in immune infiltration, gene mutations, and drug sensitivity. Notably, one of the risk genes identified by Li Jun’s team, MGST1, was also identified in our analysis. However, these studies rely heavily on algorithm-driven approaches, which limit their direct application in clinical practice. By directly associating molecular features with subtype classification, our study provides an easily applicable framework for CC risk stratification and precision treatment. This gene-based classification strategy not only enables more accurate guidance for clinical decision-making but also provides a robust basis for developing personalized therapeutic approaches tailored to different molecular subtypes. Ultimately, this approach advances the practical implementation of precision medicine in CC.

Despite the achievements of this study, several limitations remain to be addressed. First, the mechanistic exploration in this study is not sufficiently in-depth, particularly regarding the specific roles of core genes in the HRG and LRG in regulating immune responses, the TME, and tumor progression. The precise regulatory mechanisms of these genes within key molecular pathways remain unclear and require further investigation. Additionally, although suspected transient amplifying cell clusters were identified in NC group, their functional characteristics and specific connections to precancerous lesions or malignant transformation have not been thoroughly explored. This may limit a comprehensive understanding of the role of these cells in CC initiation and progression, as well as their clinical significance. These limitations provide clear directions for future research and lay the foundation for a deeper understanding of the molecular mechanisms underlying CC development.

## Conclusion

This study systematically investigates the expression patterns, biological functions, and clinical significance of NCCGs in CC and other epithelial-derived malignancies by integrating scRNA-seq and snRNA-seq, public databases, and multiple analytical approaches. By constructing comprehensive scRNA-seq and snRNA-seq atlases of CC, significant heterogeneity in the TME was revealed across different CC subtypes (e.g., SCC and ADC) and stages of progression (e.g., CCI and CCII), with dynamic changes observed in the proportional distribution of key cellular clusters, including Lym, Fb, and Ec. Using various validation methods, malignant Epi clusters and their specific signaling pathways were precisely identified, elucidating the pivotal role of NCCGs in regulating tumor cell proliferation, immune evasion, and TME remodeling. Furthermore, leveraging data from the TCGA database and external validation cohorts, this study confirmed the prognostic value of NCCGs in predicting OS and uncovered their potential applications in assessing immune infiltration, TMB, and responses to immunotherapy and chemotherapy.

Based on these findings, our study highlights the potential of NCCGs as biomarkers for predicting patient outcomes and responses to specific treatments, such as immunotherapy and chemotherapy. The identification of these genes and their associated signaling pathways opens new avenues for the development of targeted therapies aimed at modulating tumor cell proliferation, immune evasion, and TME remodeling. These discoveries not only enhance our understanding of the molecular mechanisms underlying CC but also provide a foundation for advancing personalized treatment strategies in clinical practice.

## Supplementary Information

Below is the link to the electronic supplementary material.

Supplementary Material 1

Supplementary Material 2

Supplementary Material 3

## Data Availability

All computational codes used in the analyses are available on GitHub at https://github.com/Wjw15757872931/NCCGs.

## Abbreviations

CC: Cervical cancer
NCCGs: Nuclear-cytoplasmic consistent genes
HRG: High-risk groups
LRG: Low-risk groups
HDI: Human Development Index
SCC: Cervical squamous cell carcinoma
ADC: Cervical adenocarcinomas
NC: Normal control
HPV: Human papillomavirus
scRNA-seq: Single-cell RNA sequencing
snRNA-seq: Single-nucleus RNA sequencing
TMB: Tumor mutation burden
RNA-seq: RNA sequencing
CNV: Copy number variation
DEGs: Differentially expressed genes
GO: Gene ontology
KEGG: Kyoto encyclopedia of genes and genomes
GSEAGene: Set enrichment analysis
ssGSEA: Single-sample gene set enrichment analysis
irGSEA: Iterative ranking-based gene set enrichment analysis
ESTIMATE: Stromal and immune
TME: Tumor microenvironment
TIICs: Tumor-infiltrating immune cells
RRA: Robust rank aggregation
TIP: Tracking tumor immunophenotype
IPS: Immunophenoscore
TCIA: Cancer immunome atlas
TIDE: Tumor immune dysfunction and exclusion
GDSC: Genomics of drug sensitivity in cancer
IC50: Half-maximal inhibitory concentration
FBS: Fetal bovine serum
cDNA: Complementary DNA
Lym: Lymphocytes
Epi: Epithelial cells
Mye: Myeloid cells
Fb: Fibroblasts
Ec: Endothelial cells
Smc: Smooth muscle cells
ICI: Immune checkpoint inhibitor
CTL: Cytotoxic T lymphocyte
