# Supplementary material for: Classifying the tumor immune microenvironment in cervical cancer based on nuclear cytoplasmic consistent genes
Source: Sci Rep. 2025 Nov 28;15:42703. doi: 10.1038/s41598-025-26740-4 (PMC12663427; doi:10.1038/s41598-025-26740-4)
Supplement: Supplementary file 3 — Supplementary Material 3 [file 41598_2025_26740_MOESM3_ESM.pdf]

GeneID	HR (95% CI)	P-value
ZFP36L1	1.78 (0.8 - 3.95)	1.56e-01
VGLL4	2.1 (0.85 - 5.18)	1.09e-01
TTC3	2.74 (1.16 - 6.47)	2.18e-02
TSC22D1	1.14 (0.54 - 2.41)	7.25e-01
TPT1	1.21 (0.57 - 2.56)	6.20e-01
TPM3	1.91 (0.84 - 4.35)	1.23e-01
TMSB4X	0.46 (0.22 - 0.98)	4.35e-02
TMSB10	0.67 (0.31 - 1.46)	3.16e-01
TMBIM6	1.18 (0.54 - 2.58)	6.70e-01
TM9SF2	2.11 (0.93 - 4.79)	7.48e-02
S100A6	0.86 (0.4 - 1.83)	6.90e-01
RNF213	1.07 (0.51 - 2.25)	8.65e-01
RBM47	1.66 (0.77 - 3.59)	1.94e-01
PPP1CB	1.33 (0.62 - 2.84)	4.61e-01
PERP	1.66 (0.76 - 3.59)	2.02e-01
PDIA3	1.32 (0.62 - 2.81)	4.67e-01
PABPC1	1.55 (0.72 - 3.37)	2.66e-01
P4HB	2.25 (0.99 - 5.13)	5.24e-02
MYH9	1.54 (0.71 - 3.34)	2.77e-01
MGST1	13.49 (1.83 - 99.31)	1.07e-02
LGALS3	0.84 (0.4 - 1.76)	6.42e-01
IFI16	0.6 (0.28 - 1.28)	1.86e-01
HSPB1	0.6 (0.28 - 1.26)	1.76e-01
HSD17B4	1.66 (0.73 - 3.77)	2.28e-01
HLA-E	0.96 (0.45 - 2.01)	9.07e-01
HLA-A	0.87 (0.4 - 1.89)	7.18e-01
GFPT1	1.48 (0.7 - 3.15)	3.03e-01
GAPDH	1.56 (0.72 - 3.39)	2.63e-01
FBLN1	0.8 (0.38 - 1.69)	5.63e-01
ERGIC1	1.4 (0.65 - 2.99)	3.92e-01
ELF3	1.31 (0.61 - 2.84)	4.91e-01
DNAJC3	2.46 (1.11 - 5.45)	2.69e-02
DHRS3	1.01 (0.48 - 2.14)	9.72e-01
CD74	0.69 (0.33 - 1.46)	3.34e-01
CAPZB	1.42 (0.65 - 3.1)	3.78e-01
CANX	1.34 (0.6 - 2.96)	4.76e-01
CALR	1.23 (0.57 - 2.65)	5.89e-01
B4GALT1	1.67 (0.78 - 3.57)	1.85e-01
ATP1B1	2.2 (0.99 - 4.89)	5.20e-02
ARPC2	0.87 (0.42 - 1.84)	7.21e-01
ARGLU1	0.89 (0.42 - 1.88)	7.57e-01
ANXA4	1.03 (0.49 - 2.18)	9.30e-01
ANXA1	0.69 (0.32 - 1.46)	3.27e-01
AHCYL1	2.28 (0.97 - 5.38)	5.97e-02
ACTR3	3.16 (1.2 - 8.32)	2.01e-02

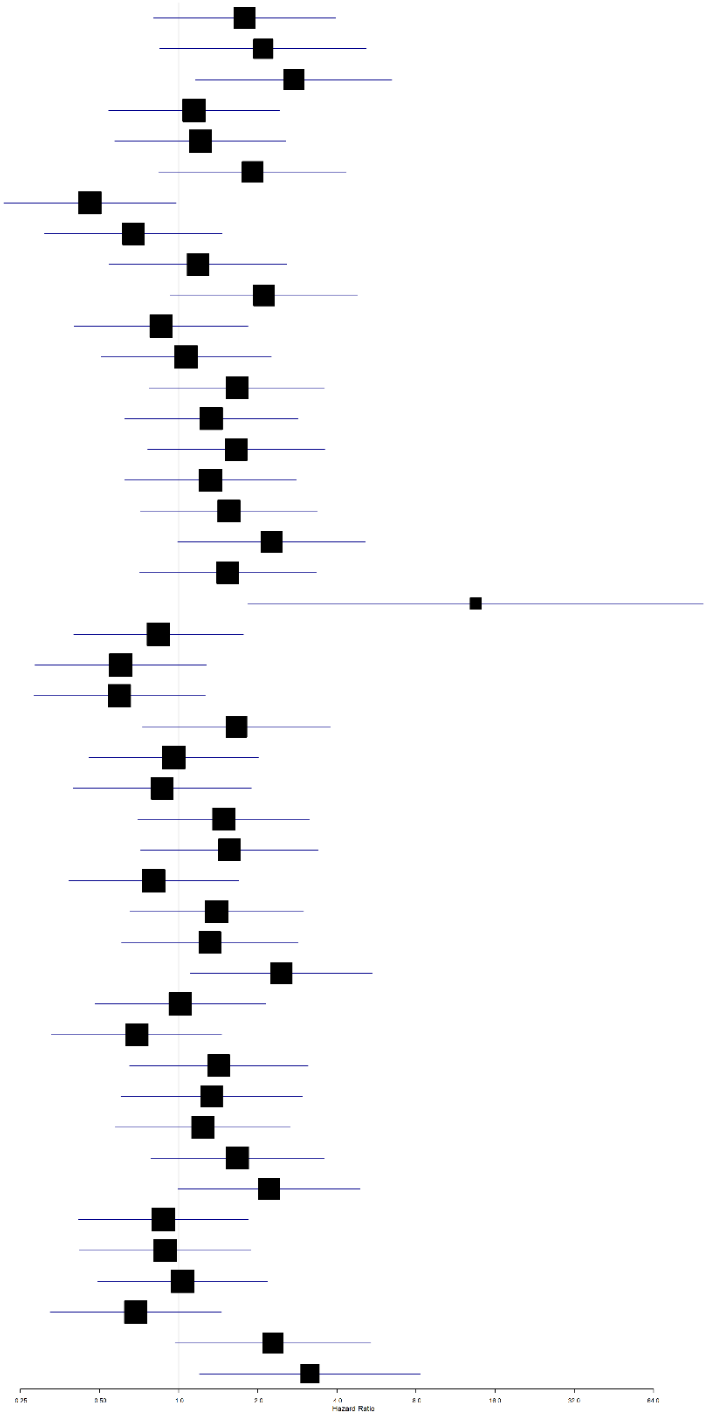

Figure S1: Forest plots showing NCCGs associated with OS in the HPV$^+$ TCGA-CESC cohort.

GeneID	HR (95% CI)	P-value
ZFP36L1	2.04 (1.01 - 4.14)	4.79e-02
VGLL4	1.9 (0.89 - 4.02)	9.55e-02
TTC3	2.12 (1.02 - 4.39)	4.28e-02
TSC22D1	1.33 (0.69 - 2.57)	3.93e-01
TPT1	2.23 (1.08 - 4.61)	3.04e-02
TPM3	2.11 (1.04 - 4.28)	3.76e-02
TMSB4X	0.95 (0.5 - 1.81)	8.74e-01
TMSB10	0.99 (0.52 - 1.88)	9.66e-01
TMBIM6	1.29 (0.66 - 2.5)	4.57e-01
TM9SF2	2.78 (1.31 - 5.89)	7.68e-03
S100A6	1.12 (0.59 - 2.14)	7.27e-01
RNF213	1.4 (0.73 - 2.71)	3.11e-01
RBM47	1.08 (0.56 - 2.06)	8.27e-01
PPP1CB	1.16 (0.6 - 2.23)	6.56e-01
PERP	1.4 (0.7 - 2.8)	3.37e-01
PDIA3	1.81 (0.92 - 3.56)	8.50e-02
PABPC1	1.39 (0.72 - 2.69)	3.22e-01
P4HB	2 (1.01 - 3.99)	4.82e-02
MYH9	1.11 (0.57 - 2.17)	7.52e-01
MGST1	4.73 (1.45 - 15.42)	9.90e-03
LGALS3	0.82 (0.43 - 1.57)	5.59e-01
IFI16	1.24 (0.63 - 2.43)	5.39e-01
HSPB1	0.87 (0.45 - 1.67)	6.72e-01
HSD17B4	1.76 (0.87 - 3.56)	1.17e-01
HLA-E	1.33 (0.67 - 2.65)	4.18e-01
HLA-A	1.47 (0.74 - 2.93)	2.74e-01
GFPT1	1.44 (0.75 - 2.76)	2.70e-01
GAPDH	1.55 (0.79 - 3.06)	2.02e-01
FBLN1	0.95 (0.5 - 1.81)	8.77e-01
ERGIC1	1.35 (0.7 - 2.59)	3.68e-01
ELF3	1.12 (0.58 - 2.16)	7.38e-01
DNAJC3	1.89 (0.97 - 3.69)	5.97e-02
DHRS3	1.61 (0.82 - 3.17)	1.67e-01
CD74	0.66 (0.35 - 1.26)	2.07e-01
CAPZB	1.66 (0.84 - 3.32)	1.47e-01
CANX	1.55 (0.78 - 3.08)	2.15e-01
CALR	1.17 (0.61 - 2.24)	6.43e-01
B4GALT1	1.26 (0.66 - 2.41)	4.90e-01
ATP1B1	1.39 (0.72 - 2.67)	3.30e-01
ARPC2	1.35 (0.68 - 2.65)	3.89e-01
ARGLU1	1.01 (0.53 - 1.94)	9.78e-01
ANXA4	1.28 (0.67 - 2.45)	4.59e-01
ANXA1	1.26 (0.64 - 2.48)	5.07e-01
AHCYL1	1.45 (0.74 - 2.85)	2.83e-01
ACTR3	2.4 (1.16 - 4.96)	1.82e-02

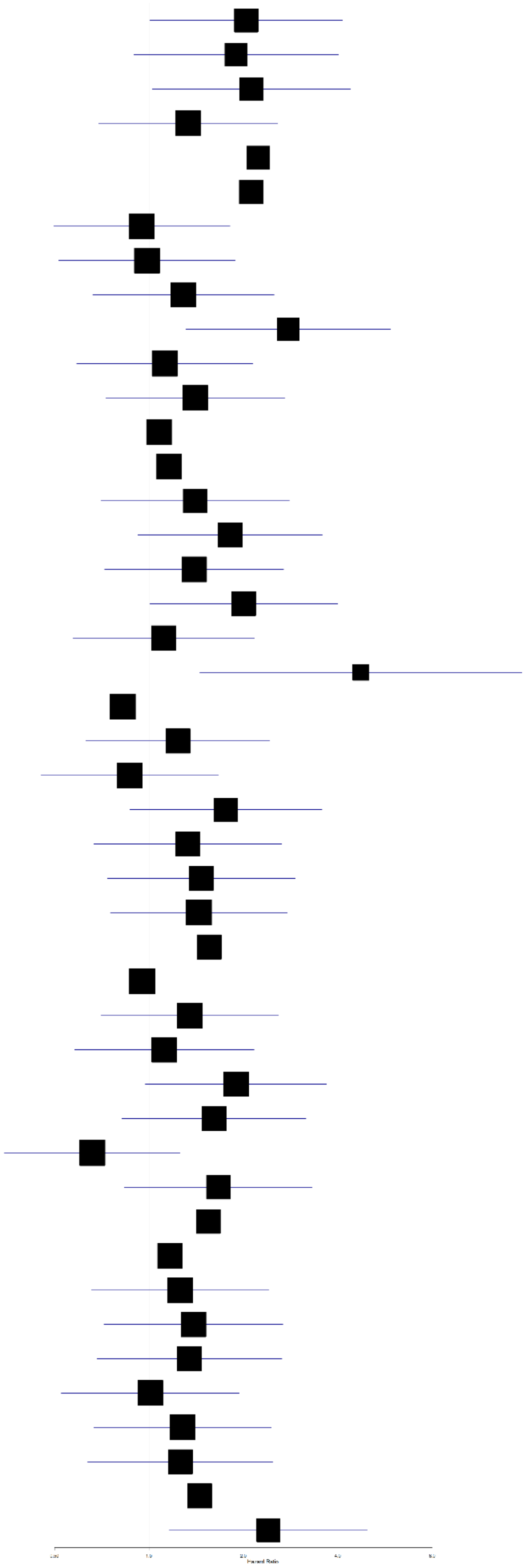

Figure S2: Forest plots showing NCCGs associated with OS in the TCGA-CESC cohort.
